# Early Infant Male Circumcision Decisions in Zambia: Demographic and Familial Influences

**DOI:** 10.1007/s10461-024-04426-8

**Published:** 2024-07-23

**Authors:** Kaylin M. Yudice, Violeta J. Rodriguez, Deborah L. Jones, Oliver Mweemba, Kasonde Bowa, Robert Zulu, Royd Kamboyi, Chloe J. Kaminsky, Stephen M. Weiss

**Affiliations:** 1 Department of Psychiatry and Behavioral Sciences, University of Miami Miller School of Medicine, Miami, FL United States of America; 2 Department of Psychology, University of Illinois Urbana Campaign, Champaign, IL United States of America; 3 Department of Health Promotion and Education, School of Public Health, University of Zambia, Lusaka, Zambia; 4 Clinical Sciences Department, University of Lusaka, Lusaka, Zambia; 5 Department of Surgery, University Teaching Hospital, Lusaka, Zambia; 6 Ministry of Health, Provincial Health Office, Ndola, Copperbelt, Zambia

**Keywords:** HIV prevention, Infant, Circumcision, Zambia

## Abstract

Public health initiatives in Zambia encourage the uptake of early infant male circumcision (EIMC) as an HIV prevention strategy. This study assessed EIMC parental decision-making during perinatal care in Lusaka, Zambia, focusing on the influence of sociodemographic factors, family, and friends. A longitudinal pilot perinatal intervention, Like Father Like Son (LFLS), was implemented among 300 couples attending antenatal clinics in four urban community health centers. Participants were assessed postpartum regarding subsequent EIMC decisions. Partners, religion, and marital status were associated with the EIMC decision-making. Large scale EIMC promotion interventions that target both parents during perinatal care should be explored.

## Introduction

Interventions by the Center for Disease Control and Prevention (CDC), the World Health Organization (WHO), and the Zambian government have been recommended to reduce HIV infection rates. One intervention widely implemented is voluntary medical male circumcision (VMMC), which reduces HIV acquisition risk by 50–70% [1]. Despite its effectiveness, VMMC initially faced low acceptance in Zambia due to varied concerns, e.g., pain and sexual abstinence [2]. The Spear and Shield (S&S) program, a VMMC promotion intervention, was highly successful in increasing VMMC in the Zambian context. Like VMMC, early infant male circumcision (EIMC) has faced challenges to uptake, despite faster healing time and long-term protection against HIV and certain STIs [3–5].

In 2017, the Zambian national level of EIMC uptake was 10%, which is low considering the procedure carries less risks than VMMC and benefits surpass the risks (200:1) [3, 6]. Acceptability of EIMC following discussion with mothers during perinatal care has been high, but actual uptake has remained low. The Zambian government has recommended demand generation strategies for EIMC to motivate parents to circumcise their male infants [7]. “Like Father, Like Son” (LFLS) is an intervention conducted in Zambia that combined demand generation strategies targeting both EIMC and VMMC.

Parents, family members, friends, and influential others may influence EIMC decision-making. Previously, components of parental EIMC decision-making pre- and post-partum were broadly examined among LFLS pilot study intervention participants [8]. The objective of the current study was to more specifically examine sociodemographic factors and the influence of extended family members and friends on parental EIMC decision-making. Given the high acceptability and low uptake of EIMC in Zambia, it was hoped this study would provide a more family-centric approach to enable insight into factors underlying the decision to circumcise male infants and serve as a foundation for future programs to stimulate EIMC uptake.

## Methods

### Ethical Considerations

Prior to study initiation, ethical approval was obtained from the University of Miami Institutional Review Board (ID: 0190354), the University of Zambia Research Ethics Committee (Ref No. 002-11-15), and the National Health Research Authority of Zambia (ID: MOD00003594). All procedures were followed in accordance with the ethical standards of the responsible committee on human experimentation (institutional and national) following the Helsinki Declaration of 1975, as revised in 2000. The parent study was registered with clinical trials.gov under trial number NCT04119414.

Written informed consent was obtained from participants before enrolling in the study. Study staff read and reviewed the informed consent document to each candidate in their preferred language in a private one-on-one session. Clarifications were made, if needed, by the study staff members. Once the staff member confirmed that the consent was understood, candidates were asked to sign or provide their mark on the consent form and the signature of a witness was obtained.

### Setting and Recruitment

The study used a longitudinal design, and information on the intervention assessments and study design have been previously described [8]. In brief, eligible participants were recruited from expecting couples attending four urban antenatal clinics in community health centers (CHCs) in Lusaka District, Zambia. Couples were eligible if they were 18 years of age or older, expecting parents, and agreed to participate as a couple. Couple members were assessed individually.

### Participants and Procedures

The sample consisted of *N* = 600 men and women (*n* = 300 couples). All women in the study were pregnant and participated with their partners. Recruited couple members provided individual written informed consent. Following consent, a baseline audio computer-assisted self-interview (ACASI) assessment was conducted in English, Nyanja, or Bemba (primary local languages) in private study offices. Participants were provided a tutorial of how to use the ACASI program by study staff. Study staff members were available to provide aid and respond to any questions as needed regarding the assessment in the participants’ preferred language.

Women and their partners were an average of 15.05 weeks pregnant and followed to 6 months postpartum. The two LFLS intervention sessions, detailed below, were deployed with couples at two months prior to delivery and immediately post-delivery. The EIMC decision-making questionnaire was deployed at 6 months postpartum.

## Measures

### Demographic Characteristics

Participants’ demographic characteristics collected included age, employment, religious affiliation, personal yearly income, education, marital status, pregnancy status, whether they had their own children or were raising others’ children, and both the mother and father’s HIV status.

### LFLS Intervention

The LFLS intervention was developed by the US and Zambia teams [9]. LFLS sessions consisted of two 90-minute meetings; session one was pre-birth, and session two was postpartum. Both sessions explored factors that might influence parental decision-making regarding EIMC; the antenatal session focused on immediate benefits of EIMC such as easier recovery and healing and cost effectiveness, while the postpartum session focused on the influence of others on EIMC decision-making. Participants had the option to invite an influential other (family or friend of the parents) to be present during the second session.

### EIMC Decision-Making Process

A survey at 6 months postpartum was used to assess the relationship between EIMC decision-making influencers, e.g., infants’ parents and extended family/friends, and EIMC uptake. The survey focused on their influence on decision-making, and assessed if the partner, partner’s mother, partner’s father, sister, uncle, grandmother, and friends were asked for an opinion on EIMC, and if they were asked, what that person recommended.

### Statistical Analyses

Univariate statistics were conducted to describe demographic characteristics and the opinion/recommendation to undergo EIMC or not between the parents and their family/friends. A series of chi-squared test of independence was employed to evaluate the relationship between both demographic factors and the influence of parents’ family and friends on the uptake of EIMC. Statistical significance was designated as *p* < 0.05. Statistical Package for the Social Sciences (SPSS) version 26 was utilized for analyses.

## Results

### Demographic Characteristics

Demographic characteristics associated with the decision to undergo EIMC include marital status (X^2^ (1, *N* = 283) = 5.106, *p* = 0.024) and religious affiliation (X^2^ (3, *N* = 283) = 9.073, *p* = 0.028). The relationship between other demographic factors and the decision to undergo EIMC are shown in Table 1.

**Table 1:** Demographic characteristics by parents combined who circumcised Versus who did not (*N* = 283)

Variables	Did not circumcise (*n* = 184)	Circumcised (*n* = 99)	X^2^, *p*
Age			
18–29 years old 30–69 years old	111 (60.3%) 73 (39.7%)	60 (60.6%) 39 (39.4%)	33.815, 0.288
Employment			
Not working/volunteering Work part-time or full-time	77 (41.8%) 107 (58.2%)	40 (40.4%) 59 (59.6%)	0.055, 0.814
Religious Affiliation			
Christian Jehovah’s witness Muslims Other	130 (70.7%) 13 (17.1%) 0 (0.0%) 41 (22.3%)	77 (77.8%) 3 (3.0%) 3 (3.0%) 16 (16.2%)	9.073, 0.028
Personal Income (Year)			
< 5000 Kwacha (∼ USD$256) 5000 Kwacha or Greater	133 (72.3%) 51 (27.7%)	80 (80.8%) 19 (19.2%)	2.513, 0.113
Education			
No schooling/primary school Secondary or tertiary school	50 (27.2%) 134 (72.8%)	34 (34.3%) 65 (65.7%)	1.585, 0.208
Marital Status			
Single/Separated/Divorced Married	28 (15.2%) 156 (84.8%)	6 (6.1%) 93 (93.9%)	5.106, 0.024
Children			
No Yes	64 (34.8%) 120 (65.2%)	28 (28.3%) 71 (71.7%)	1.239, 0.266
Raising Others’ Children			
No Yes	102 (55.4%) 82 (44.6%)	45 (45.5%) 54 (54.5%)	2.568, 0.109
Children in the Home			
No Yes	47 (25.5%) 137 (74.5%)	16 (16.2%) 83 (83.8%)	3.274, 0.070
Mother’s HIV Status			
Does not have HIV Has HIV Hasn’t told partner	152 (82.6%) 19 (10.3%) 13 (7.1%)	86 (86.9%) 9 (9.1%) 4 (4.0%)	1.219, 0.544
Father’s HIV Status			
Does not have HIV Has HIV Not previously tested for HIV	162 (88.0%) 20 (10.9%) 2 (1.1%)	83 (83.8%) 12 (12.1%) 4 (4.0%)	2.869, 0.238
Father’s MC Status			
Circumcised Not circumcised	40 (44.0%) 51 (56.0%)	16 (32.0%) 34 (68.0%)	1.927, 0.165

### Decision-Making Processes

Decision-making of both mothers and fathers are outlined in Table 2. Notably, partners’ suggestions were associated with the decision to undergo EIMC for both mothers (X^2^ (2, *N* = 125) = 17.760, *p* = < 0.001) and fathers (X^2^ (2, *N* = 125) = 22.297, *p* = < 0.001). Of infants who were circumcised, 90.0% of parents made that decision together, indicating collaborative decision-making among the sample. In terms of external influence, i.e. family and friends, while parents did tend to seek suggestion from family, there appeared to be no influence on the decision to circumcise the infant.

**Table 2:** Parental decision making who circumcised Versus who did not (*N* = 125)

Variables	Did not circumcise (*n* = 84)		Circumcised (*n* = 41)		X^2^, *p*	
**Mothers**						
Partner Factors						
Decision					5.252, 0.072	
Alone	1 (11.1%)		3 (7.5%)			
Partner	2 (22.2%)		1 (2.5%)			
Together	6 (66.7%)		36 (90.0%)			
Did not cause a Serious Disagreement	69 (82.1%)		36 (92.3%)		2.203, 0.138	
Asked for Partner’s opinion	75 (89.3%)		40 (97.6%)		2.564, 0.109	
Partner suggested EIMC	46 (61.3%)		39 (97.5%)		17.760, < 0.001	
Immediate Family Members						
Sister suggested EIMC	28 (82.4%)		15 (100.0%)		3.016, 0.221	
Mother suggested EIMC	31 (73.8%)		17 (94.4%)		4.147, 0.126	
Father suggested EIMC	30 (85.7%)		13 (92.9%)		1.291, 0.524	
Extended Family and Friends						
Uncle (mother’s brother) suggested EIMC	21 (91.3%)		9 (81.8%)		2.168, 0.338	
Grandmother suggested EIMC	18 (64.3%)		11 (100.0%)		5.283, 0.071	
Friend suggested EIMC	29 (72.5%)		18 (100.0%)		6.109, 0.047	
Another person suggested EIMC	26 (74.3%)		19 (90.5%)		2.524, 0.283	
**Fathers**						
Partner Factors						
Decision						1.050, 0.591
Alone		2 (28.6%)		6 (15.0%)		
Partner		0 (0.0%)		2 (5.0%)		
Together		5 (71.4%)		32 (80.0%)		
Did not cause a Serious Disagreement		68 (80.0%)		39 (97.5%)		6.758, 0.009
Asked for Partner’s opinion		80 (94.1%)		41 (97.6%)		0.766, 0.382
Partner suggested EIMC		48 (60.0%)		41 (100.0%)		22.297, < 0.001
Immediate Family Member						
Sister suggested EIMC		23 (88.5%)		12 (85.7%)		2.041, 0.360
Mother suggested EIMC		23 (85.2%)		9 (90.0%)		0.398, 0.819
Father suggested EIMC		22 (88.0%)		10 (100.0%)		1.313, 0.252
Extended Family and Friends						
Uncle (mother’s brother) suggested EIMC		20 (100.0%)		7 (100.0%)		n/a
Grandmother suggested EIMC		20 (90.9%)		5 (71.4%)		3.515, 0.172
Friend suggested EIMC		31 (81.6%)		12 (92.3%)		1.501, 0.472
Another person suggested EIMC		29 (80.6%)		10 (76.9%)		3.034, 0.219

### EIMC Rates

The rate of EIMC in previous research following an informational intervention directed only to mothers was 11% [6]. The LFLS intervention, addressing both parents, identified a rate of 35% for EIMC uptake among participating couples [8]. Out of 150 couples who met the criteria to participate, fifty-two had their newborn son circumcised [8].

## Discussion

This study explored the influence of demographic and interpersonal factors on EIMC decisions. The results of this study offer insight into the decision-making process surrounding EIMC in Zambia, revealing its collaborative nature.

### Demographic Characteristics and their Influence

Participants were predominantly young, employed, and identifying as Christian with modest income by Zambian standards, and reflected a broad cross-section of Zambian society. Notably, the decisions regarding EIMC were not influenced by other factors, e.g., age, employment status, income, or education. Previous research has found education, specifically no schooling, to be associated with a higher chance of EIMC [7]. The current study did not find this association, such that the decision to circumcise excluded this factor. In contrast, the influence of religious affiliation and marital status on the decision to circumcise indicated that cultural and religious values, as well as family structure, may play a role in the EIMC decision-making processes.

### Decision-Making Dynamics among Parents

While the father is historically the primary decision maker in the family in Zambia, this study presents evidence that the mother has an influence. In fact, couples engaged in a predominantly collaborative decision-making process with a high level of agreement and a minimum of serious disagreements [10]. Both partners’ suggestions to circumcise their infant had an impact on the final decision to undergo EIMC. These results illustrate the role of mutual influence and spousal support in family health-related decisions. As such, the interdependent nature of partners’ opinions within mother and father dyads is an important foundation for implementation of future EIMC intervention strategies [11].

### The Role of Extended Family and Social Networks

While mothers and fathers asked both extended and immediate family for their suggestions on EIMC, others’ suggestions to circumcise the infant did not appear to impact mothers’ and fathers’ final decision to undergo EIMC. Compared to the impact of partners’ suggestions to undergo EIMC, extended family and friends did not appear to have an impact on the decision to circumcise infants. As such, it appears that immediate family members had less influence over EIMC decisions compared to the parental unit.

### Implications for Future Interventions

Study findings suggest strategies for future interventions aiming to increase EIMC rates, should address both parents, recognizing the collaborative nature of their decision-making process. Additionally, the influence of religious affiliation and marital status should be considered when implementing interventions. While the influence of extended family was not significant in this study, their role should not be discounted. Results may differ in other culture groups. As both mothers and fathers reach out to family members for health-related advice, broader familial engagement could support increased awareness and acceptance of EIMC.

### Limitations and Future Research

The main limitation of this study is the location of the study, which was conducted in urban Lusaka, Zambia. Participants were predominantly from an urban region, and results should not be generalized to other communities and rural regions of Zambia or other sub-Saharan African countries. Future studies should be conducted across a larger geographic and cultural scale to understand if decision-making differs by region or culture. Another limitation includes barriers associated with the COVID-19 pandemic, which increased reluctance for parents to bring their infants to the clinic and may have reduced social contact. Finally, there was a cholera outbreak in the community, further increasing clinic limitations in participation. These barriers may have limited uptake of EIMC.

## Conclusion

This study assessed the influence of family members and friends on parents’ EIMC decision-making. Results suggest that while demographic characteristics such as religion and marital status influenced the decision-making process, the strongest influence was the partners’ recommendation. The partner’s opinion is a key determinant in the decision to elect for an infant to undergo EIMC. Findings suggest that the influence of family members and friends are less pronounced than the collaborative decision-making of the parental unit.

Though there are challenges in promoting EIMC in Zambia, this study sheds light on the importance of engaging partners and understanding their influence on the decision-making process. Increasing awareness and education about EIMC, along with partner involvement, may contribute to the success of interventions in Zambia and ultimately reduce the prevalence of HIV and some STIs. However, future research and interventions should consider the cultural and regional variations within Zambia and the potential impact of external factors on healthcare decision-making.

## References

[1] Weiss SM, Zulu R, Jones DL, Redding CA, Cook R, Chitalu N. The Spear and Shield intervention to increase the availability and acceptability of voluntary medical male circumcision in Zambia: a cluster randomised controlled trial. Lancet HIV. 2015;2(5):e181–9. 26120594 10.1016/S2352-3018(15)00042-9 PMC4478609

[2] Hatzold K, Mavhu W, Jasi P, Chatora K, Cowan FM, Taruberekera N, et al. Barriers and motivators to voluntary medical male circumcision uptake among different age groups of men in Zimbabwe: results from a mixed methods study. PLoS ONE. 2014;9(5):e85051. 24802746 10.1371/journal.pone.0085051 PMC4011705

[3] Morris BJ, Kennedy SE, Wodak AD, Mindel A, Golovsky D, Schrieber L, et al. Early infant male circumcision: systematic review, risk-benefit analysis, and progress in policy. World J Clin Pediatr. 2017;6(1):89. 28224100 10.5409/wjcp.v6.i1.89 PMC5296634

[4] Zambia Statistics Agency MoHZ, ICF. Zambia demographic and health survey 2018. Lusaka, Zambia, and Rockville. Maryland, USA: Zambia Statistics Agency, Ministry of Health, and ICF; 2019.

[5] Sgaier SK, Sharma S, Eletskaya M, Prasad R, Mugurungi O, Tambatamba B, et al. Attitudes and decision-making about early-infant versus early-adolescent male circumcision: demand-side insights for sustainable HIV prevention strategies in Zambia and Zimbabwe. PLoS ONE. 2017;12(7):e0181411. 28749979 10.1371/journal.pone.0181411 PMC5531536

[6] Kamanga SP. Uptake of early infant medical male circumcision as an HIV prevention intervention in Chongwe district. Zambia: The University of Zambia; 2017.

[7] Rodriguez VJ, Weiss SM, Hernández L, Bowa K, Zulu R, Jones DL. Zambian parents’ perspectives on early-infant Versus early-adolescent male circumcision. AIDS Behav. 2023:1–7. 10.1007/s10461-022-03912-1 PMC10338018 36692607

[8] Weiss SM, Rodriguez VJ, Cook RR, Bowa K, Zulu R, Mweemba O, et al. Increasing early infant male circumcision uptake in Zambia: like father like son. PLoS ONE. 2023;18(8):e0289819. 37561707 10.1371/journal.pone.0289819 PMC10414584

[9] Mweemba O, Rodriguez VJ, Jones DL, Desgraves JF, Msimuko R, Mofya R et al. Factors influencing neonatal male circumcision uptake among expecting couples in Zambia: formative findings. AIDS Care. 2023:1–8. 10.1080/09540121.2023.2223900 PMC13334836 37408444

[10] Mavhu W, Hatzold K, Ncube G, Fernando S, Mangenah C, Chatora K, et al. Unpacking early infant male circumcision decision-making using qualitative findings from Zimbabwe. BMC Int Health Hum Rights. 2017;17:1–7. 28069002 10.1186/s12914-016-0111-1 PMC5223435

[11] Jones D, Cook R, Arheart K, Redding CA, Zulu R, Castro J, et al. Acceptability, knowledge, beliefs, and partners as determinants of Zambian men’s readiness to undergo medical male circumcision. AIDS Behav. 2014;18:278–84. 23757123 10.1007/s10461-013-0530-0 PMC3815686

